# Antibiotic Resistance and Virulence Determinants of *Pseudomonas aeruginosa* Isolates Cultured from Hydrocarbon-Contaminated Environmental Samples

**DOI:** 10.3390/microorganisms13030688

**Published:** 2025-03-19

**Authors:** Chioma Lilian Ozoaduche, Balázs Libisch, Daniel Itoro, Iyore Blessing Idemudia, Katalin Posta, Ferenc Olasz

**Affiliations:** 1 Agribiotechnology and Precision Breeding for Food Security National Laboratory, Institute of Genetics and Biotechnology, Hungarian University of Agriculture and Life Sciences, 2100 Gödöllő, Hungary; ozoaduche.chioma.lilian@phd.uni-mate.hu (C.L.O.); posta.katalin@uni-mate.hu (K.P.); olasz.ferenc.gyorgy@uni-mate.hu (F.O.); 2 Doctoral School of Biology, Hungarian University of Agriculture and Life Sciences, 2100 Gödöllő, Hungary; 3 Sustainable Environment Development Initiative (SEDI), Benin City 300102, Nigeria; 4 Microbiology Research Laboratory, University of Benin, Benin City 300283, Nigeria; dannypraiz14@gmail.com; 5 Department of Microbiology, University of Benin, Benin City 300283, Nigeria; iyore.idemudia@uniben.edu

**Keywords:** antibiotic resistance, efflux pump, hydrocarbon pollution, *mexR*, One Health, solvent tolerance

## Abstract

Crude oil and its derivates are among the most important environmental pollutants, where *P. aeruginosa* strains producing AlkB1 and AlkB2 alkane hydroxylases are often involved in their biodegradation. The aim of this study was to analyze antibiotic resistance and virulence determinants of a *P. aeruginosa* isolate cultured from a hydrocarbon-contaminated soil sample from Ogoniland, Nigeria, and to compare its characteristics with *P. aeruginosa* isolates cultured worldwide from hydrocarbon-contaminated environments or from clinical samples. Using the ResFinder reference database, a *catB7* chloramphenicol acetyltransferase gene, an *ampC*-type PDC β-lactamase gene, and an OXA-50 type β-lactamase gene were identified in all *P. aeruginosa* strains analyzed in this study. In some of these *P. aeruginosa* strains, loss-of-function mutations were detected in the regulatory genes *mexR*, *nalC*, or *nalD*, predicting an efflux-mediated acquired antibiotic-resistance mechanism. Several *P. aeruginosa* sequence types that were associated with oil-contaminated environments have also been cultured from human clinical samples worldwide, including sequence types ST532, ST267, ST244, and ST1503. Our findings also indicate that environmental *P. aeruginosa* may serve as the source of human infections, warranting further studies from a One Health perspective about the application of *P. aeruginosa* for the in situ bioremediation of hydrocarbon-contaminated sites.

## 1. Introduction

Crude oil and its derivates are among the most important environmental pollutants resulting from industrial activities, where the global annual spillage of petroleum contaminants has been estimated to exceed 1.2 million tons [1,2,3]. Crude oil production contributes about 6% to the Nigerian GDP, and its Ogoni area has been yielding oil since 1957 [4,5]. The settlements of Ebubu and Bomu hosted the first oil wells in Ogoniland [6]. Environmental oil pollution has been documented in this region for decades, with a substantial effect also on the Ejama-Ebubu village [7,8,9], with contaminations permeating deep into the subsurface [9]. Ogoniland has a high annual precipitation, and oil spills may therefore be carried away across farmland [10,11]. Crops and other plants can suffer stress when oil enters their root zone. Investigations recorded significant residual oil pollution in the Ejama-Ebubu community despite several attempts at cleanup [12].

In the Ejama-Ebubu community, Rivers State, Nigeria, culturable hydrocarbon utilizing bacteria and fungi were obtained from aged, oil-impacted soil [7]. Akani and colleagues assessed oil-impacted fresh water swamp vegetation in Ejama-Ebubu in Rivers State and revealed higher values of total heterotrophic bacterial (THB) and saprophytic fungal counts during the wet season than in the dry season [13]. A variety of microorganisms were found in the soil from the Ejama-Ebubu oil-spill site, where the high concentration of aerobic hydrocarbon-utilizing bacteria was related to the degree of oil pollution [14]. Bacteria like *Alcaligenes faecalis*, *Bacillus cereus*, *Chromobacterium* spp., *Flavobacterium* spp., *Pseudomonas* spp., *Citrobacter* spp., *Enterobacter* spp., and *Micrococus* spp. and fungi such as *Aspergillus niger*, *A. fumigatus*, *A. flavus*, *A. aculeatus*, *Penicillium citrinum*, *Fusarium* spp., *Rhizopus* spp., *Microsporum canis*, and *Acremonium* sp. as well as yeasts have been isolated from the oil-polluted Ebubu-Ejama community [7,13,14,15].

*Pseudomonas* spp. strains are frequently isolated from hydrocarbon-contaminated soils, particularly in its first phase of biodegradation, although in certain cases, Gram-positive bacteria have been found to be most abundant [16,17,18]. Among these bacteria, *P. aeruginosa* is a species cultured often from hydrocarbon-impacted environments [19,20]. The Gram-negative bacterium *P. aeruginosa* is metabolically highly versatile, allowing it to inhabit numerous ecological niches in addition to soil and aquatic environments. This versatility allows *P. aeruginosa* to be an opportunistic pathogen, colonizing the respiratory tract of cystic fibrosis patients or causing various nosocomial infections, and it is also an important environmental bacterium degrading ecological pollutants such as detergents or *n*-alkanes [21,22,23].

Despite the isolation and whole-genome sequencing of several *P. aeruginosa* strains from oil-polluted environments worldwide [24,25], their antibiotic resistance and virulence determinants and their clonal relatedness to *P. aeruginosa* clinical isolates have not yet been specifically examined by a genomics-based analysis. The aim of the current study was to specifically investigate the antibiotic resistance and virulence determinants of a *P. aeruginosa* isolate cultured from hydrocarbon-contaminated soil in Nigeria by a whole-genome sequencing approach and to compare it with other *P. aeruginosa* isolates from clinical samples and from hydrocarbon-impacted environments of a worldwide distribution; herein also lies the novel aspect of the present study.

## 2. Materials and Methods

### 2.1. Isolation and Identification of P. aeruginosa Strain CHA1

**Figure 1:**
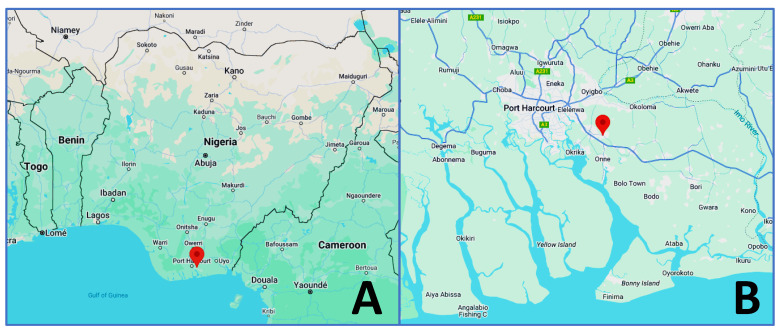
The geographical location of the crude oil-polluted site within Nigeria (**A**) and its local area (**B**) where *P. aeruginosa* strain CHA1 was isolated. The corresponding locations are indicated by red points on the map.

*P. aeruginosa* strain CHA1 was isolated in July 2023 from a crude oil-polluted soil sample collected in the Ejama-Ebubu community in the Eleme Local Government Area of Rivers State, Nigeria, with the latitude and longitude of 4.790018 N and 7.152437 E, respectively (Figure 1). Its phenotypic characteristics included rod-shaped cells subjected to microscopic examination, yellow-colored colonies on *Pseudomonas* cetrimide agar, and blue-green-colored colonies on nutrient agar. An isolated colony was identified by PCR amplification and Sanger sequencing of the 16S rRNA gene (Biomi Ltd., Gödöllő, Hungary) using the universal primers 27F 5′-AGAGTTTGATCCTGGCTCAG-3′ and 1492R 5′-GGTTACCTTGTTACGACTT-3′ [26].

### 2.2. Whole-Genome Sequencing of P. aeruginosa Strain CHA1

*P. aeruginosa* strain CHA1 was subjected to whole-genome sequencing (WGS) by Biomi Ltd. (Gödöllő, Hungary) on Illumina MiSeq platform using 2 × 250 bp paired-end reads. De novo contig-level assembly of the sequencing data was performed by the SPAdes v. 3.15.4 assembler, at 149× genome coverage, and the contig-level draft genome assembly was submitted to the NCBI Genomes database under project PRJNA1041298. Further bioinformatic tools available on the Center for Genomic Epidemiology (CGE) platform were applied for a WGS-based characterization of isolate CHA1, including ResFinder 4.1 [27], the in silico serotyping of *P. aeruginosa* isolates [28], MobileElementFinder v1.0.3 [29], and KmerFinder v3.2 [30].

### 2.3. Detection of Acquired Antibiotic-Resistance Genes

Acquired antibiotic-resistance genes (ARGs) were also searched for in WGS data by the ABRicate v1.0.1 tool [31,32] against the ResFinder 4.1 database [27] with the settings of ≥80% threshold for sequence identity [33], and of minimum coverage ≥80%. Translated ORFs were searched by the BLASTP tool against the NCBI Protein database v5. The ABRicate v1.0.1 tool using the PasmidFinder database version 2021-Mar-27 and MobileElementFinder v.1.0.3 did not detect the presence of plasmids in *P. aeruginosa* strains listed in Table 1.

### 2.4. Searching for Genomic Mutations Causing an Antibiotic-Resistant Phenotype

Amino acid substitutions in the proteins encoded by the following genes were searched for by comparisons with the corresponding sequence of the *P. aeruginosa* PAO1 reference strain [34,35,36]: *ampC*, *ampR*, *mexR*, *mexS*, *mexZ*, *nalC*, *nalD*, and *dacB* [37,38,39]. The translated amino acid sequences of the corresponding ORFs of the PAO1 reference strain were used to search for mutations potentially conferring an antibiotic-resistant phenotype in *P. aeruginosa*.

**Table 1:** *P. aeruginosa* environmental and reference strains analyzed in this study.

Nr.	Strain Code	Country	Sampling Location	Sample Type	NCBI Biosample	Ref.
1	CHA1	Nigeria	Ogoniland, Rivers State	crude oil contaminated soil	SAMN38280573	this work
2	PA1-Petro	Brazil	Oilfield in State of Sergipe	oil production water	SAMN20156365	[40]
3	CMIP 8.1	Brazil	Rio de Janeiro	crude oil well	SAMN18912870	[41]
4	W-101	China	Dagang Oil Reservoir	sewage	SAMN09767472	[42]
5	2K-1	Peru	Talara Oil Refinery	oil-contaminated environment	SAMN29360594	[43]
6	6K-11	Peru	Talara Oil Refinery	oil-contaminated environment	SAMN29360595	[43]
7	ATCC33988	USA	Ponca City, OK	fuel storage tank	SAMN02767933	[44]
8	M8A1	Colombia	Caño Limon Oilfield	crude oil residual water	SAMN04916455	[25]
9	IMP66	China	Daqing Oilfield	crude oil	SAMN08915456	[25]
10	ATCC27853	USA	Boston Hospital	human blood culture	SAMN29939566	[45]
11	M8A4	Colombia	Caño Limon Oilfield	crude oil residual water	SAMN04916454	[25]
12	DQ8	China	Daqing Oilfield	crude oil polluted soil	SAMN02470947	[25]
13	L6-1	China	Xinjiang Oilfield	oil reservoir production fluid	SAMN04325380	[46]
14	8D	China	Ansai Oilfield	oilfield production water	SAMN27926083	[47]
15	PAO1	Australia	Melbourne	human wound sample	SAMN02603714	[34,35,36]

Besides the *P. aeruginosa* CHA1 strain, other *P. aeruginosa* isolates with publicly available WGS data in the NCBI databases were also involved in this analysis (Table 1), where the corresponding NCBI BioSample IDs are listed in Table 1.

### 2.5. In Vitro Antibiotic Susceptibility Testing of Strain CHA1

The in vitro antibiotic susceptibility of *P. aeruginosa* strain CHA1 was tested by the disc diffusion and broth dilution methods according to EUCAST [48]. The impact of the efflux-pump inhibitor phenylalanine-arginine β-naphthylamide (PAβN) (Sigma-Aldrich, Saint Louis, MO, USA) was tested by adding PAβN at the concentration of 50 mg/L to Mueller–Hinton Broth [49,50] in a broth microdilution assay to assess the contribution of efflux pumps to the phenotypic antibiotic resistance of strain CHA1. The phenotypic effect of efflux pumps was considered significant if the MICs in the absence of PAβN were at least 4-fold higher than the MICs in the presence of PAβN [50,51].

### 2.6. Detection of P. aeruginosa Virulence Genes

The WGS data of the examined *P. aeruginosa* isolates were screened using the ABRicate tool [31,32] for *P. aeruginosa* virulence genes against the VFDB database version 2021-Mar-27 [52] at ≥80% coverage and ≥80% identify values. The prevalence of virulence factors and ARGs among hydrocarbon-impacted and clinical isolates was determined by the Kruskal–Wallis test and by Pearson correlation analyses using IBM SPSS Statistics v29 software (IBM SPSS Inc., Chicago, IL, USA). Multivariate clustering of the isolates based on the detected virulence determinants was performed using the paired group (UPGMA) algorithm using PAST 4.08 software (Natural History Museum, University of Oslo, https://www.nhm.uio.no/english/research/resources/past/, accessed on 31 October 2021).

### 2.7. Protein Sequence Alignment of AlkB1 and AlkB2 Alkane Hydroxylases

Alignment of the encoded protein sequences for the AlkB1 and AlkB2 alkane hydroxylases of strain CHA1 with those of the reference *P. aeruginosa* strain PAO1 were performed using the Clustal Omega Multiple Sequence Alignment (MSA) version 1 tool on the platform of the EMBL’s European Bioinformatics Institute (https://www.ebi.ac.uk/, accessed on 31 October 2021).

### 2.8. Construction of Phylogenetic Trees from Whole-Genome Sequence Data

The reference sequence alignment-based phylogeny builder (REALPHY) [53] was applied to infer a phylogenetic tree from the whole-genome sequence data of the *P. aeruginosa* strains summarized in Table 1 and the additional clinical *P. aeruginosa* isolates that shared their MLST sequence type with some of the environmental *P. aeruginosa* isolates (see Supplementary Table S1). In this analysis, all provided WGS sequences were mapped to the selected reference genome of the PAO1 *P. aeruginosa* strain via bowtie2.

### 2.9. Assessment of the Contribution of P. aeruginosa Efflux Pumps to Hexane Tolerance

*P. aeruginosa* strains CHA1 and ATCC 27853 were grown in 96-well sterile microplates where the wells contained a concentration range of 0% to 90% hexane in Mueller–Hinton Broth (Oxoid, Basingstoke, UK). The impact of efflux-pump inhibitor PAβN (Sigma-Aldrich, Saint Louis, MO, USA) was tested by adding PAβN at a concentration of 50 mg/L to the growth medium [49,50] in a broth microdilution assay to assess the contribution of efflux pumps to the hexane-tolerance of strains CHA1 and ATCC 27853. The microplates were inoculated with a bacterial suspension of 0.5 McFarland density and incubated in ambient air at 37 °C for 24 h. Bacterial growth was measured at OD600 in a Boeco BMR-100 microplate reader (Hamburg, Germany).

## 3. Results

### 3.1. Characterization of P. aeruginosa Strain CHA1 Cultured from Hydrocarbon-Polluted Soil of the Ejama-Ebubu Community in Nigeria

The details of the isolation and identification of *P. aeruginosa* strain CHA1 are described in the Materials and Methods Section. Initial biochemical tests showed that isolate CHA1 was oxidase-, catalase-, and citrate-positive but indole- and urease-negative. Its 16S rRNA gene sequence was identical to that of the reference *P. aeruginosa* strain ATCC 27853. Whole-genome sequencing and bioinformatic analyses showed that *P. aeruginosa* isolate CHA1 was a sequence type ST1503 serotype O1 *P. aeruginosa* isolate. The Abricate tool using the PlasmidFinder version 2021-Mar-27 database and MobileElementFinder v.1.0.3 [29,31] did not detect plasmids in the contig-level draft genome assembly of strain CHA1.

In the course of the biodegradation of *n*-alkanes by *P. aeruginosa*, the first oxygenation step is catalyzing by integral-membrane AlkB alkane hydroxylases [54,55]. The CHA1 genome contained 1-1 copies for the chromosomally located *alkB1* and *alkB2* alkane hydroxylase genes, respectively. Supplementary Figure S1 shows alignments of the respective protein sequences with AlkB1 and AlkB2 of *P. aeruginosa* strain PAO1. The AlkB1 and AlkB2 proteins of the two strains showed 99.4% and 100% identities, respectively, with no amino acid substitutions in the eight catalytically essential conserved histidine residues [54,55], inferring that the encoded AlkB enzymes of strain CHA1 are also functional. On the other hand, a *P. putida* GPo1-like additional *alkB* alkane hydroxylase gene [54,55] was not present in the draft genome of strain CHA1, as opposed to some other *P. aeruginosa* isolates, such as strains CIMP8.1, DQ8, and PA1-Petro (Table 1).

### 3.2. In Vitro Antibiotic Susceptibility of P. aeruginosa Strain CHA1

Strain CHA1 displayed consistent antibiotic susceptibility patterns when tested by the disk diffusion and broth microdilution methods according to EUCAST [48] (Table 2). The isolate showed intermediate resistance, according to current EUCAST breakpoints, to most tested β-lactam antibiotics with the exception of meropenem. Intermediate (I) resistance is applied when bacteria are in vitro inhibited by a concentration of an antimicrobial agent that is associated with an uncertain therapeutic effect [48].

The efflux-pump inhibitor phenylalanine-arginine β-naphthylamide (PAβN) [49] was used at the concentration of 50 mg/L in Mueller–Hinton broth in a microdilution assay according to [50,51], and ≥8-fold decreases in the MICs for ceftazidime, trimethoprim, and chloramphenicol were found in the presence of 50 mg/L PAβN (see Table 3), confirming the contribution of antibiotic efflux pump(s) to the observed in vitro antibiotic susceptibility pattern of *P. aeruginosa* strain CHA1 [49,50,51,56]. In vitro antibiotic susceptibility profiles were not published for the other whole-genome-sequenced hydrocarbon-impacted *P. aeruginosa* strains listed in Table 1, and the strains were not available for susceptibility testing. Therefore, the current study focused on the analyses of these isolates based on their WGS data.

**Table 2:** In vitro antibiotic susceptibility pattern of *P. aeruginosa* strain CHA1 ^a^.

Testing Method		PIT	CTZ	CEP	IMI	MER	CIP	GEN	TOB	CHL	COL
Disk diffusion	Inhibitory zone (mm) ^b^	27	20	20	26	25	34	19	23	0	-
	Interpretation	I	I	R	I	S	I		S	R	
Broth microdilution	MIC (mg/L)	4	2	1	2	1	0.25	0.5	0.5	64	0.5
	Interpretation	I	I	I	I	S	I	WT ^c^	S	R	S

^a^ Abbreviations for antibiotics: PIT, piperacillin-tazobactam; CTZ, ceftazidime; CEP, cefepime; IMI, imipenem; MER, meropenem; CIP, ciprofloxacin; GEN, gentamicin; TOB, tobramycin; CHL, chloramphenicol; COL, colistin. ^b^ Diameters of inhibitory zones are provided in mm, with the following interpretations: S, susceptible; I, intermediate; R, resistant. ^c^ WT stands for wildtype according to the 8 mg/L epidemiological cutoff published by EUCAST for GEN against *P. aeruginosa* [48].

**Table 3:** Changes in MIC values in the presence of 50 mg/L PAβN for strain CHA1.

Antibiotic	MIC (mg/L)		
	Ceftazidime	Trimethoprim	Chloramphenicol
−PAβN ^a^	2	128	64
+PAβN ^a^	≤0.25	≤8	≤2
Fold-change in MIC	≥8	≥16	≥32

^a^ Abbreviations: −PAβN, no PAβN was added; +PAβN, PAβN was added.

### 3.3. Assessment of ARGs and Known Genetic Mutations Leading to Antibiotic Resistance in Hydrocarbon-Impacted and Reference P. aeruginosa Strains

The WGS data of *P. aeruginosa* strains isolated from hydrocarbon-impacted environments listed in Table 1 were analyzed for resistance genes (ARGs) as detected by ResFinder v4.1 and for genetic mutations (Table 4) in the respective genes of *ampC*, *ampR*, *mexR*, *mexS*, *mexZ*, *nalC*, *nalD*, and *dacB* that would potentially contribute to antibiotic resistance in *P. aeruginosa* isolates [37,38,39]. Table 4 shows that most isolates cultured from hydrocarbon-impacted environments had a similar ARG profile, including *catB7*, a chloramphenicol acetyltransferase gene found in *P. aeruginosa* [57]; *crpP*, a previously suspected ciprofloxacin-modifying enzyme with contradictory results on its function [58]; an *ampC*-type class C PDC β-lactamase gene [59]; and an OXA-50 type β-lactamase that confers decreased susceptibility to ampicillin, ticarcillin, and meropenem in *P. aeruginosa* [60]. Furthermore, strain DQ8 also carried an *aph(3′)-Ia (aphA7)* aminoglycoside phosphotransferase gene [61]. Among *ampR*, *mexR*, *mexS*, *mexZ*, *nalC*, *nalD*, and *dacB* regulatory genes in which certain type of mutations may lead to acquired antibiotic resistance in *P. aeruginosa* [37,38,39], several amino acid substitutions compared to the corresponding protein sequence of the *P. aeruginosa* strain PAO1 were found (Table 4). Mutations that were proposed to be natural mutations among *P. aeruginosa* isolates are indicated by green color in Table 4 [39]. In addition to mutations resulting in certain amino acid substitutions, frameshift mutations (FM) in *ampR* (strain IMP66), *mexR* (strain 8D), and *nalC* (PA1-Petro) were also identified, while *nalD* was not detected in strain 6K-11 (see Table 4).

### 3.4. Phylogenetic Analysis Based on the Whole-Genome Sequence Data

**Figure 2:**
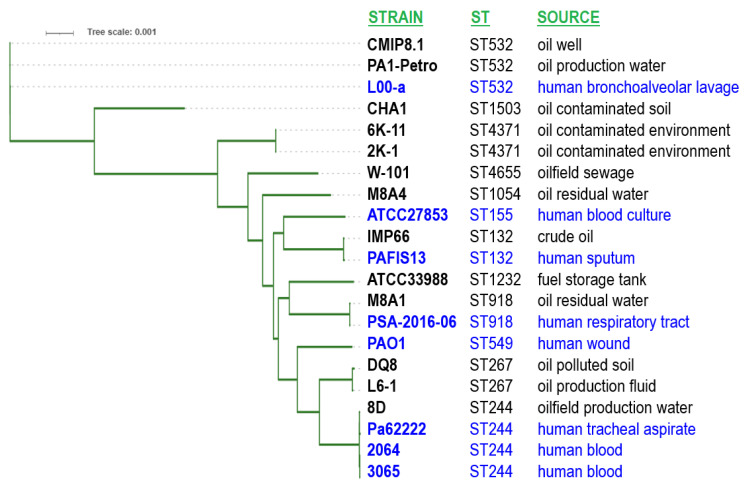
Phylogenetic tree inferred by REALPHY for clinical and environmental *P. aeruginosa* strains. Blue color indicates human clinical isolates, while ST numbers provide MLST sequence types. The tree scale is indicated in the upper left corner.

Figure 2 displays the phylogenetic tree inferred by REALPHY using the WGS data of *P. aeruginosa* strains summarized in Table 1 and the clinical *P. aeruginosa* isolates given in Supplementary Table S1. Clustering of the isolates based on their WGS data correlated well with their 7-gene MLST sequence type.

**Table 4:** ARGs identified by ResFinder v4.1 at ≥80% coverage and amino acid substitutions detected in the products *of ampC*, *ampR*, *mexR*, *mexS*, *mexZ*, *nalC*, *nalD*, and *dacB* compared to that in strain PAO1. FM stands for frameshift mutations and PDC16-L for PDC-16-like. ST stands for the sequence type of the isolates as determined by in silico multi-locus sequence typing (MLST) using the Center for Genomic Epidemiology (CGE) platform (https://www.genomicepidemiology.org/, accessed on 31 October 2021). In every strain listed in this table, an additional *fosA* and an *aph(3′)-IIb* gene were also detected.

Strain Code	Country	ST	Sero-Type	OXA-50 Family Variant	*catB7*	*crpP*	*aph(3′)-Ia*	*ampC*							*ampR*						*mexR*		*mexS*			*mexZ*	*nalC*				*nalD*	*dacB*	
								PDC-1	PDC-3	PDC-5	PDC-8	PDC16-L	PDC-59	PDC-120	*E114A*	*G283E*	*M288R*	*R119C*	*R244W*	*FM*	*V126E*	*FM*	*D249N*	*A75V*	*E278D*	*L128M*	*G71E*	*S209R*	*A145V*	*FM*	*deletion*	*A394P*	*A474T*
PA1-Petro	Brazil	ST532	O11	OXA-906																													
CMIP 8.1	Brazil	ST532	O11	OXA-906																													
CHA1	Nigeria	ST1503	O1	OXA-1032																													
W-101	China	ST4655	O1	OXA-494-like																													
2K-1	Peru	ST4371	O11	OXA-494-like																													
6K-11	Peru	ST4371	O11	OXA-494-like																													
ATCC33988	USA	ST1232	O11	OXA-50																													
M8A1	Colombia	ST918	O6	OXA-50-like																													
IMP66	China	ST132	O6	OXA-494																													
ATCC27853	USA	ST155	O6	OXA-396																													
M8A4	Colombia	ST1054	O6	OXA-396																													
DQ8	China	ST267	O5	OXA-1026																													
L6-1	China	ST267	O5	OXA-1026																													
8D	China	ST244	O5	OXA-847																													
PAO1	Australia	ST549	O5	OXA-50																													

### 3.5. Comparing the Prevalence of Virulence and Antibiotic-Resistance Genes Between Clinical and Environmental Isolates

The *P. aeruginosa* virulence determinants detected by the VFDB reference database among the examined clinical and environmental strains are shown in Supplementary Table S2, while those for ARGs detected by ResFinder are given in Supplementary Table S3. The identified virulence and antibiotic-resistance determinants did not show a significantly different distribution and prevalence between environmental versus clinical isolates based on individual genes or based on the total number of virulence factors detected for the tested strains (*p* > 0.05). Supplementary Figure S3 shows the clustering of the analyzed *P. aeruginosa* isolates based on their identified virulence factors, which provided several similar patterns in clustering together the isolates that were related to each other also by their sequence types (STs) and WGS-based phylogenetic analysis.

### 3.6. Contribution of P. aeruginosa Efflux Pumps to Hexane Tolerance

The efflux-pump inhibitor PAβN had a marked impact on the hexane tolerance of both the CHA1 and ATCC 27853 strains. In the absence of PAβN, both *P. aeruginosa* strains could tolerate hexane and grow in the presence of hexane, however, with a decreasing level of OD600 values reached after 24 h of growth as the percentage of hexane increased in the Mueller–Hinton broth medium. Furthermore, in the presence of 50 mg/L PAβN, the 40% and higher proportions of hexane in the broth medium caused a >99% inhibition in growth for both *P. aeruginosa* strains CHA1 and ATCC 27853. 

## 4. Discussion

**Figure 3:**
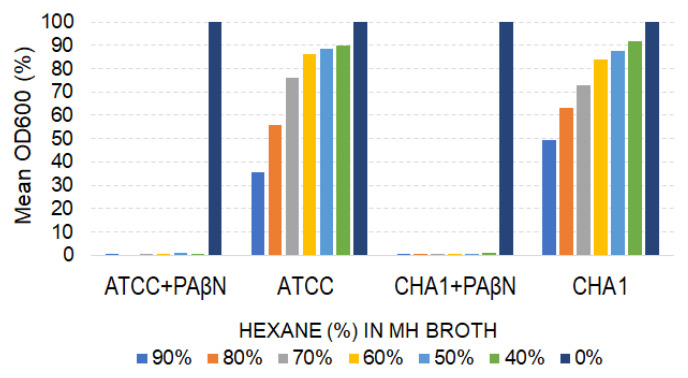
Effect of PAβN on growth of *P. aeruginosa* strains in the presence of hexane. Mean OD600 values (% of control) were recorded after 24 h of growth. The colors indicate the percentage of hexane content of the Mueller–Hinton Broth. +PAβN indicates 50 mg/L PAβN added to the growth medium. ATCC and CHA1 stand for *P. aeruginosa* strains ATCC27853 and CHA1, respectively.

Microbial communities in crude oil-impacted habitats are often dominated by *Pseudomonas* spp., which exhibit survival advantages over other bacterial genera [25]. Competition between hydrocarbon-assimilating microbes is an important factor affecting the bioremediation of oil-polluted sites through their adaptation and survival in the hydrocarbon-enriched environment [62]. Efflux-mediated extrusion of toxic compounds is one of the adaptive mechanisms in *P. aeruginosa*, where Mex-type efflux systems are involved as a major mechanism of hydrocarbon resistance by the extrusion of toxic hydrocarbons from the bacterial cells (Figure 3) [63]. The solvent-tolerant K1261 and K1262 strains of *P. aeruginosa* obtained by serial passages in media containing increasing concentrations of hexane showed an increased expression of *mexAB*-*oprM* and had elevated MICs against antibiotics such as carbenicillin, cefepime, ciprofloxacin, tetracycline, and chloramphenicol [64]. Furthermore, the increased expression of *mexCD-oprJ* induced by *n*-hexane in *P. aeruginosa* strain K1542 caused elevated MICs against norfloxacin and erythromycin [65].

Overexpression of the MexAB-OprM system caused by mutation(s) in genes involved in its regulation (such as in *mexR*, *nalC*, *or nalD*) can increase the MICs against several β-lactam antibiotics, including meropenem [66]. The MexAB-OprM pump displays a broad substrate profile, and thus, its mutational overexpression can lead to resistance against β-lactam antibiotics (except imipenem) and to resistance to other antibiotics, including quinolones, tetracyclines, and macrolides [67]. It has been described that genetic events such as frameshift mutations (FM), disruptions, or premature stops, which lead to loss of functionality of *nalC*, *nalD* or *mexR*, are expected to up-regulate the *mexAB-oprM* operon [68]. MexS is, on the other hand, a suppressor of MexT (an activator of the multidrug efflux system MexEF-OprN), and thus, mutations in MexS can also cause multidrug-resistance in *P. aeruginosa* [69,70]. In the presence of *n*-alkanes, therefore, the blocking of these efflux pumps can lead to cell toxicity, as has also been shown for aromatic hydrocarbons [24,63,71].

Concerning the amino acid substitutions found in the regulatory proteins MexR and NalC in the environmental *P. aeruginosa* strain CHA1 (Table 4), similar substitutions were also reported, for example, in the *P. aeruginosa* clinical strain HUMV_110, where an analogous combination of mutations was detected (MexR: Val-126Glu; NalC: Gly-71Glu, and Ser-209Arg) (see also Supplementary Figure S2), where strain HUMV_110 also possessed an additional premature stop codon in *mexZ* [68]. The ^126^valine → glutamic acid substitution (V126E) was found to be associated with the overproduction of MexAB by real-time reverse-transcription PCR [72]. Likewise, the amino acid substitution ^209^Ser → Arg (S209R) in NalC was shown by real-time reverse-transcription PCR to be associated with *mexA* gene relative expression levels at least five times that of the control strain [73] (see Supplementary Figure S2). The above amino acid substitutions were also linked to multidrug-resistant and carbapenem-resistant phenotypes in these *P. aeruginosa* isolates, respectively [72,73].

A ≥32-fold decrease was observed in the chloramphenicol MIC of strain CHA1 in the presence of 50 mg/L PAβN and ≥8-fold decrease for the MICs against ceftazidime and trimethoprim (Table 3), indicating the presence of an efflux-mediated resistance mechanism in strain CHA1.

Some inconsistencies were reported in the results of different laboratory methods for the in vitro susceptibility testing of *P. aeruginosa* against cefepime, where five isolates were assigned to the intermediate group by microdilution and the resistant group by an automated system, and one isolate was susceptible to cefepime by microdilution and resistant by disk-diffusion. Furthermore, two other strains were susceptible to cefepime by microdilution and intermediate by disk-diffusion. These observations suggest that testing by the disc diffusion method in certain cases can assign *P. aeruginosa* to a higher level of resistance against cefepime compared to the microdilution method [68]. A comparable case can be observed for the susceptibility of strain CHA1 against cefepime according to the EUCAST interpretation guidelines (Table 2) [48].

Similar to the MexAB-OprM efflux pump, overexpression of MexXY is common (at about 10 to 30) among *P. aeruginosa* clinical isolates and causes decreased susceptibility to aminoglycosides and cefepime [69,74]. However, MexCD-OprJ overexpression is uncommon among clinical isolates of *P. aeruginosa*, with the notable exception of those from cystic fibrosis [68]. Loss-of-function mutations (such as complete deletion or FMs) in *mexR*, *nalC*, and *nalD* were reported to be associated with the hyperproduction of the MexAB-OprM efflux pump [39]. Therefore, the FM detected in *mexR* of strain 8D and *nalC* of PA1-Petro and the deletion of *nalD* in strain 6K-11 (see Table 4) is expected to be associated with the overexpression of *mexAB-oprM*.

Antibiotic resistance on crude oil-contaminated sites may emerge as a result of the acquisition of *n*-alkane tolerance, where organic solvent-tolerant mutants of *P. aeruginosa* can be selected with increased MICs against β-lactams, fluoroquinolones, chloramphenicol, tetracycline, and/or novobiocin [24,75]. Mutations, for example, in *mexR*, the repressor gene of the *mexAB-oprM* efflux operon, were identified in solvent-tolerant mutant *P. aeruginosa* strains, where the MexAB-OprM system was found to be the far superior efflux system providing solvent tolerance in *P. aeruginosa* compared to other efflux systems [24,75]. These and other observations on the distribution of multidrug-resistant *P. aeruginosa* isolates between different habitats [32,76] also highlight the need for a One Health approach to uncover the possible role of environmental reservoirs in the epidemiology and spread of multidrug-resistant *P. aeruginosa* between different hosts and environments. It was also proposed that unconventional oil and gas extraction may create hotspots for antimicrobial resistance [77]. Antimicrobial agents, specifically biocides, are used in unconventional oil and gas extraction to mitigate microbially induced corrosion and gas souring, which might potentially also co-select for antibiotic-resistance mechanisms in the impacted bacteria [77].

Regarding ARGs identified using the ResFinder reference database, a *catB7* chromosome-encoded chloramphenicol acetyltransferase gene, an *ampC*-type class C PDC β-lactamase gene, an OXA-50 type β-lactamase gene, a *fosA* fosfomycin thiol transferase, and an *aph(3′)-IIb* a chromosomal-encoded aminoglycoside phosphotransferase gene were demonstrated in all *P. aeruginosa* strains analyzed in this study (Table 4), while *crpP*, a previously suspected ciprofloxacin-modifying enzyme, was present in only 9 of the analyzed 15 isolates in Table 4. The *bla*_PDC_ and *bla*_OXA-50_ family member genes are important and intrinsic ARGs and are expected to be present in *P. aeruginosa* [78]. An additional APH(3′)-Ia aminoglycoside phosphotransferase encoding gene was identified in isolate DQ8, suggesting its strain-specific acquisition by horizontal gene transfer. The *aph(3′)-Ia* gene of strain DQ8 is located on a contig of 1186 nucleotides, which shows 99.77% identity in a 867 bp region with transposon Tn*1412* from *P. aeruginosa* strain 2293E [79]; however, other sections of Tn*1412* were not detected in strain DQ8. The acquisition of antibiotic-resistance determinants by *P. aeruginosa* through horizontal gene transfer (HGT) at crude oil-contaminated sites may be enhanced by the highly mutagenic polycyclic aromatic hydrocarbons (PAHs) [24,80,81]. Hydrocarbon pollution can enhance HGT through selective pressure, as resistance to various antibiotics might be a selective advantage for *P. aeruginosa* isolates to maintain sufficient levels of cell surface hydrophobicity and attachment to hydrocarbon substrates in polluted environments [24,82,83]. The spread of ARGs in PAH-contaminated environments was suggested to also be enhanced by co-selection when several types of contaminants are present in the soil microbiome and also by the reduction in microbial communities in soils contaminated with hydrocarbons, where such soils could be more sensitive to the introduction and spread of ARGs [80,84].

The ampC-type PDC β-lactamases and the OXA-50-type β-lactamases of *P. aeruginosa* are considered as naturally encoded *P. aeruginosa* β-lactamase enzymes, which are in general an inducible cephalosporinase and a constitutively expressed oxacillinase, respectively [59,60]. Therefore, HGT is not expected to be associated with their identification in the examined isolates, similar to other detected chromosome-encoded ARGs, including *aph(3′)-IIb* and *catB7*. However, the PDC-3 and PDC-5 variants detected in several of the *P. aeruginosa* isolates in Table 4 encode enzymes that were found to have significantly increased catalytic efficiencies against cefepime and imipenem compared to PDC-1 [59]. In a global analysis, *bla*_PDC-16_ was one of most region-specific PDC alleles; namely, it was the second most common in Sub-Saharan Africa but only the 5th–13th most common in other regions, consistent with the detection of its variant in strain CHA1 from Nigeria [78].

Furthermore, the OXA-494 enzyme (see Table 4) presented an increased activity against ticarcillin compared to OXA-50 [85], and OXA-396 was found among the most frequent OXA-50 variants and was widely distributed among *P. aeruginosa* strains from patients with bronchiectasis [86].

Clonal analysis based on the 7-gene MLST scheme [87] revealed that the ST532 sequence type that was assigned to isolates PA1-Petro and CMIP 8.1 (Table 4) was also detected among clinical *P. aeruginosa* isolates, and it was associated with MDR or XDR phenotypes that include the production of horizontally-acquired β-lactamases [76,86]. The ST1503 sequence type assigned to isolate CHA1 from Nigeria (Table 4) was also found among *P. aeruginosa* isolated from children in Mexico who developed bacteremia [88] and among *P. aeruginosa* isolates colonizing cystic fibrosis patients [89]. An ST918 *P. aeruginosa* strain was cultured from a respiratory tract sample at a tertiary care center in Cologne, Germany, and thus shared its sequence type with that of strain M8A1 from Columbia (Table 4) [90]. The ST132 sequence type of isolate IMP66 from China (Table 4) has been reported from clinical isolates in Croatia and the Czech Republic, too [91,92], while ST267 of isolates DQ8 and L6-1 (Table 4) were found among clinical *P. aeruginosa* strains in Tunisia [93] and drinking water from China [94]. Lastly and most notably, ST244 of isolate 8D from China (Table 4) is considered a high-risk epidemic *P. aeruginosa* global clinical clone [76,95,96,97].

These observations further support that environmental *P. aeruginosa* may serve as the source of human infections, pointing to the need for additional assessments of the potential public health risks associated with the application of *P. aeruginosa* for the in situ bioremediation of hydrocarbon-contaminated sites. Such future studies would be indeed justified, as *P. aeruginosa* offers valuable potential in the remediation of organic pollutants, including in heavy oil-, diesel-, and kerosene-polluted water bodies [98]. Analyses of bacterial communities of oil-contaminated sites showed that petroleum pollution can cause a decrease in the relative abundances of a range of soil bacteria with the concurrent enrichment of hydrocarbon-degrading strains such as *Pseudomonas* spp., thereby modifying the composition of the dominant soil microbial community [24].

In conclusion, we isolated and characterized a *P. aeruginosa* strain from hydrocarbon-impacted soil in Nigeria and compared its features with other *P. aeruginosa* strains of a global distribution. The novel aspect of the current study was that it specifically investigated by a WGS approach the antibiotic resistance and virulence determinants of *P. aeruginosa* isolates from hydrocarbon-contaminated environments and also their clonal relatedness with clinical *P. aeruginosa* strains. Besides some ARGs commonly carried by *P. aeruginosa* strains, loss-of-function mutations in relevant regulatory genes predicting efflux-mediated resistance mechanisms were also found. Our data clearly demonstrate that several *P. aeruginosa* sequence types (STs) that were detected in oil-contaminated environmental samples have also been isolated from human clinical samples worldwide. Therefore, further studies are needed to explore from a One Health perspective the antibiotic-resistance mechanisms of environmental *P. aeruginosa* isolates and possible ways for their dissemination into the human population. These further studies shall be conducted on a *P. aeruginosa* strain collection where phenotypic in vitro antibiotic-susceptibility testing and other culture-based characterization of the isolates can be directly linked and combined with their WGS-based molecular analyses.

## Data Availability

The original contributions presented in the study are included in the article, and further inquiries can be directed to the corresponding author. The contig-level draft genome assembly of *P. aeruginosa* strain CHA1 was submitted to the NCBI Genomes database under project PRJNA1041298 (https://www.ncbi.nlm.nih.gov/home/genomes/, accessed on 1 March 2025).

## Supplementary Materials

The following supporting information can be downloaded at https://www.mdpi.com/article/10.3390/microorganisms13030688/s1, Figure S1: Alignment of the protein sequences of AlkB1 and AlkB2 enzymes of *P. aeruginosa* strains CHA1 and PAO1; Figure S2: Multiple alignments of the protein sequences encoded by the *mexR* and *nalC* genes of *P. aeruginosa* isolates; Figure S3: Clustering of *P. aeruginosa* isolates based on virulence factors detected in WGS data; Table S1: Recent *P. aeruginosa* clinical isolates included in the phylogenetic analysis; Table S2: *P. aeruginosa* virulence factors detected in clinical and environmental isolates; Table S3: ARGs detected by ResFinder in *P. aeruginosa* strains.
