# Supplementary material for: Antibiotic Resistance and Virulence Determinants of Pseudomonas aeruginosa Isolates Cultured from Hydrocarbon-Contaminated Environmental Samples
Source: Microorganisms. 2025 Mar 19;13(3):688. doi: 10.3390/microorganisms13030688 (PMC11945736; doi:10.3390/microorganisms13030688)

Supplemenentary Table S1. Recent *P. aeruginosa* clinical isolates included in the phylogenetic analysis

Strain	MLST Sequence Type	Accession of Assembly or SRA	NCBI Sample Number	Location	Host	Sample
P. aeruginosa PAFIS13_49	ST132	GCA_046356665.1	SAMN35344029	Madrid, Spain	Human	Sputum
P. aeruginosa 2064	ST244	GCA_043354405.1	SAMN39333833	Houston, USA	Human	Blood
P. aeruginosa 3065	ST244	GCA_043354405.1	SAMN39333833	Houston, USA	Human	Blood
P. aeruginosa Pa62222	ST244	GCA_030410985.1	SAMN31178863	Moscow, Russia	Human	Tracheal aspirate
P. aeruginosa L00-a	ST532	GCA_023520735.1	SAMN28206123	Chicago, USA	Human	Bronchoalveolar lavage
P. aeruginosa PSA-2016-06	ST918	ERR5490542	SAMEA8270516	Cologne, Germany	Human	Respiratory tract

Supplementary Table S2. *P. aeruginosa* virulence factors detected in clinical and environmental isolates

Coverage of VF detected (% of reference)

Isolate	MLST Sequence Type	Group	Total number of VFs detected	*alg44*	*alg8*	*algA*	*algB*	*algC*	*algD*	*algE*	*algF*	*algG*	*algI*	*algJ*	*algK*	*algL*	*algP/ algR3*	*algQ*	*algR*	*algU*	*algW*	*algX*	*algZ*	*aprA*	*chpA*	*chpB*	*chpC*	*chpD*
PAFIS13_49	ST132	clinical	225	100	100	100	100	100	100	100	100	100	100	100	100	100	98,87	100	100	100	100	100	100	100	100	100	100	100
IMP66	ST132	environmental	227	100	100	100	100	100	100	100	100	100	100	100	100	100	98,87	100	100	100	100	100	100	100	100	100	100	100
ATCC27853	ST155	clinical	226	100	100	100	100	100	100	100	100	100	100	100	100	100	100	100	100	100	100	100	100	100	100	100	100	100
8D	ST244	environmental	240	100	100	100	100	100	100	100	100	100	100	100	100	100	100	100	100	100	99,83	100	100	100	100	100	100	100
2064	ST244	clinical	227	100	100	100	100	100	100	100	100	100	100	100	100	100	0	100	100	100	99,83	100	100	100	100	100	100	100
3065	ST244	clinical	227	100	100	100	100	100	100	100	100	100	100	100	100	100	0	100	100	100	99,83	100	100	100	100	100	100	100
Pa62222	ST244	clinical	235	100	100	100	100	100	100	100	100	100	100	100	100	100	100	100	100	100	99,83	100	100	100	100	100	100	100
L6-1	ST267	environmental	240	100	100	100	100	100	100	100	100	100	100	100	100	100	97,73	100	100	100	100	100	100	100	100	100	100	100
DQ8	ST267	environmental	233	100	100	100	100	100	100	100	100	100	100	100	100	100	81,78	100	100	100	100	100	100	100	100	99,81	0	100
PA1-Petro	ST532	environmental	218	100	100	100	100	100	100	100	100	100	100	100	100	100	98,87	100	100	100	100	100	100	100	99,92	100	100	100
CMIP8,1	ST532	environmental	217	100	100	100	100	100	100	100	100	100	100	100	100	100	98,87	100	100	100	100	100	100	100	99,92	100	100	100
L100-a	ST532	clinical	218	100	100	100	100	100	100	100	100	100	100	100	100	100	98,87	100	100	100	100	100	100	100	99,92	100	100	100
PAO1	ST549	clinical	241	100	100	100	100	100	100	100	100	100	100	100	100	100	100	100	100	100	100	100	100	100	100	100	100	100
PSA-2016-06	ST918	clinical	234	100	100	100	100	100	100	100	100	100	100	100	100	100	100	100	100	100	99,83	100	100	100	100	100	100	99,87
M8A1	ST918	environmental	230	100	100	100	100	100	100	100	100	100	100	100	100	100	100	100	100	100	99,83	100	100	100	100	100	100	99,87
M8A4	ST1054	environmental	224	100	100	100	100	100	100	100	100	100	100	100	100	100	100	100	100	100	100	100	100	100	100	100	100	100
ATCC33988	ST1232	environmental	224	100	100	100	100	100	100	99,86	100	94,85	83,3	100	100	100	100	100	100	100	100	100	100	100	100	100	100	100
CHA1	ST1503	environmental	231	100	100	100	100	100	100	100	100	100	100	100	100	100	98,87	100	100	100	100	100	100	100	99,92	100	100	100
2K-1	ST4371	environmental	217	100	100	100	100	100	100	100	100	100	100	100	100	100	95,18	100	100	100	100	100	100	100	99,92	100	100	100
6K-11	ST4371	environmental	174	0	0	0	100	100	0	0	0	0	0	0	0	0	95,18	100	100	100	100	0	100	100	99,92	100	100	100
W-101	ST4655	environmental	229	100	100	100	100	100	100	100	100	100	100	99,74	100	100	100	100	100	100	100	100	100	100	100	100	100	100

Isolate	*chpE*	*clpV1*	*dotU1*	*exoS*	*exoT*	*exoU*	*exoY*	*exsA*	*exsB*	*exsC*	*exsD*	*exsE*	*fha1*	*fimT*	*fimU*	*fimV*	*fleI/flag*	*fleN*	*fleP*	*fleQ*	*fleR*	*fleS*	*flgA*	*flgB*
PAFIS13_49	100	100	100	100	100	0	100	100	100	100	100	100	0	100	100	100	0	100	0	100	100	100	100	100
IMP66	100	100	100	100	100	0	100	100	100	100	100	100	100	100	100	100	0	100	0	100	100	100	100	100
ATCC27853	100	100	100	100	100	0	100	100	100	100	100	100	99,2	100	100	100	0	100	0	100	100	100	100	100
8D	100	100	100	100	100	0	100	100	100	100	100	100	100	100	100	100	100	100	100	100	100	100	100	100
2064	100	100	100	100	100	0	100	100	100	100	100	100	0	100	100	100	100	100	100	100	99,2	100	100	100
3065	100	100	100	100	100	0	100	100	100	100	100	100	0	100	100	100	100	100	100	100	99,2	100	100	100
Pa62222	100	100	100	100	100	0	100	100	100	100	100	100	100	100	100	100	100	100	100	100	100	100	100	100
L6-1	100	100	100	100	100	0	100	100	100	100	100	100	100	100	100	100	100	100	100	100	100	100	100	100
DQ8	100	100	100	100	100	0	100	100	100	100	100	100	98,8	100	100	100	100	100	100	99,86	100	100	100	100
PA1-Petro	100	100	100	0	100	100	100	100	100	100	100	100	100	0	0	100	0	100	0	100	100	100	100	100
CMIP8,1	100	100	100	0	100	100	100	100	100	100	100	100	100	0	0	100	0	100	0	100	100	100	100	100
L100-a	100	100	100	0	100	100	100	100	100	100	100	100	99,6	0	0	100	0	100	0	100	100	100	100	100
PAO1	100	100	100	100	100	0	100	100	100	100	100	100	100	100	100	100	100	100	100	100	100	100	100	100
PSA-2016-06	100	100	100	100	100	0	100	100	100	100	100	100	100	100	100	99,96	100	100	100	100	100	100	100	100
M8A1	100	100	100	100	100	0	100	100	100	0	100	100	99,53	100	100	100	100	100	100	100	100	100	100	100
M8A4	100	100	100	100	100	0	100	100	100	100	100	100	98,39	100	100	100	0	100	0	100	100	100	100	100
ATCC33988	100	100	0	100	100	0	100	100	100	100	100	100	100	100	100	100	100	100	100	100	100	100	100	100
CHA1	100	100	100	0	100	100	100	100	100	100	100	100	100	100	100	100	0	100	0	100	100	100	100	100
2K-1	100	100	100	100	100	0	100	100	100	100	100	100	98,8	0	0	100	0	100	0	100	100	100	100	100
6K-11	100	100	100	100	100	0	100	100	100	100	100	100	98,8	0	0	100	0	100	0	100	100	100	100	100
W-101	100	100	100	100	100	0	100	100	100	100	100	100	99,6	100	100	100	100	100	100	100	100	100	100	100

Isolate	*flgC*	*flgD*	*flgE*	*flgF*	*flgG*	*flgH*	*flgI*	*flgJ*	*flgK*	*flgL*	*flgM*	*flgN*	*flhA*	*flhB*	*flhF*	*fliA*	*fliC*	*fliD*	*fliE*	*fliF*	*fliG*	*fliH*	*fliI*	*fliJ*	*fliK*	*fliL*	*fliM*	*fliN*	*fliO*
PAFIS13_49	100	100	100	100	100	100	100	100	99,71	0	100	100	100	100	100	100	0	0	100	100	100	100	100	100	100	100	100	100	100
IMP66	100	100	100	100	100	100	100	100	99,71	0	100	100	100	100	100	100	0	0	100	100	100	100	100	100	100	100	100	100	100
ATCC27853	100	100	100	100	100	100	100	100	99,71	0	100	100	100	100	100	100	0	0	100	100	100	100	100	100	100	100	100	100	100
8D	100	100	100	100	100	100	100	100	100	100	100	100	100	100	100	100	100	100	100	100	100	100	100	100	100	100	100	100	100
2064	100	100	100	100	100	100	100	100	100	100	100	100	100	100	100	100	100	100	100	100	100	100	100	100	100	100	100	100	100
3065	100	100	100	100	100	100	100	100	100	100	100	100	100	100	100	100	100	100	100	100	100	100	100	100	100	100	100	100	100
Pa62222	100	100	100	100	100	100	100	100	100	100	100	100	100	100	100	100	100	100	100	100	100	100	100	100	100	100	100	100	100
L6-1	100	100	100	100	100	100	100	100	100	100	100	100	100	100	100	100	100	100	100	100	100	100	100	100	100	100	100	100	100
DQ8	100	100	100	100	100	100	100	100	100	100	100	100	100	100	100	100	100	100	100	100	100	100	100	100	100	100	100	100	100
PA1-Petro	100	100	100	100	100	100	100	100	99,71	0	100	100	100	100	100	100	0	0	100	100	100	100	100	100	100	100	100	100	100
CMIP8,1	100	100	100	100	100	100	100	100	99,71	0	100	100	100	100	100	100	0	0	100	100	100	100	100	100	100	100	100	100	100
L100-a	100	100	100	100	100	100	100	100	99,71	0	100	100	100	100	100	100	0	0	100	100	100	100	100	100	100	100	100	100	100
PAO1	100	100	100	100	100	100	100	100	100	100	100	100	100	100	100	100	100	100	100	100	100	100	100	100	100	100	100	100	100
PSA-2016-06	100	100	100	100	100	100	100	100	100	100	100	100	100	100	100	100	100	100	100	100	100	100	100	100	100	100	100	100	100
M8A1	100	100	100	100	100	100	100	100	100	100	100	100	100	100	100	100	100	100	100	100	100	100	100	100	100	100	100	100	100
M8A4	100	100	100	100	100	100	100	100	99,71	0	100	100	100	100	100	100	0	0	100	100	100	100	100	100	100	100	100	100	100
ATCC33988	100	100	100	100	100	100	100	100	100	100	100	100	100	100	100	100	100	100	100	100	100	100	100	100	100	100	100	100	100
CHA1	100	100	100	100	100	100	100	100	99,71	0	100	100	100	100	99,77	100	0	0	100	100	100	100	100	100	100	100	100	100	100
2K-1	100	100	100	100	100	100	100	100	99,71	0	100	100	100	100	100	100	0	0	100	100	100	100	100	100	100	100	100	100	100
6K-11	100	100	100	100	100	100	100	100	99,71	0	100	100	100	100	100	100	0	0	100	100	100	100	100	100	100	100	100	100	100
W-101	100	100	100	100	100	100	100	100	100	100	100	100	100	100	99,77	100	100	100	100	100	100	100	100	100	100	100	100	100	100

Isolate	*fliP*	*fliQ*	*fliR*	*fliS*	*fptA*	*fpvA*	*hcp1*	*hsiA1*	*hsiB1/vipA*	*hsiC1/vipB*	*hsiE1*	*hsiF1*	*hsiG1*	*hsiH1*	*hsiJ1*	*icmF1/tssM1*	*lasA*	*lasB*	*lasI*	*lip1*	*mbtH-like*	*motA*	*motB*
PAFIS13_49	100	100	100	0	100	0	100	100	100	100	100	100	100	100	100	100	100	100	100	100	100	100	100
IMP66	100	100	100	0	100	0	100	100	100	100	100	100	100	100	100	100	100	100	100	100	100	100	100
ATCC27853	100	100	100	0	100	0	100	100	100	100	100	100	100	100	100	100	100	100	100	100	100	100	100
8D	100	100	100	100	100	100	100	100	100	100	100	100	100	100	100	100	100	100	100	100	100	100	100
2064	100	100	100	100	100	100	100	100	100	100	100	100	100	100	100	100	100	100	100	100	100	100	100
3065	100	100	100	100	100	100	100	100	100	100	100	100	100	100	100	100	100	100	100	100	100	100	100
Pa62222	100	100	100	100	100	100	100	100	100	100	100	100	100	100	100	100	100	100	100	100	100	100	100
L6-1	100	100	100	100	100	100	100	100	100	100	100	100	100	100	100	100	100	100	100	100	100	100	100
DQ8	100	100	99,36	100	100	0	100	100	100	100	100	100	100	100	100	99,97	100	100	100	100	100	100	100
PA1-Petro	100	100	100	0	100	0	100	100	100	100	100	100	100	100	100	100	100	100	100	100	100	100	100
CMIP8,1	100	100	100	0	100	0	100	100	100	100	100	100	100	100	100	100	100	100	100	100	100	100	100
L100-a	100	100	100	0	100	0	100	100	100	100	100	100	100	100	100	100	100	100	100	100	100	100	100
PAO1	100	100	100	100	100	100	100	100	100	100	100	100	100	100	100	100	100	100	100	100	100	100	100
PSA-2016-06	100	100	100	100	100	0	100	100	100	100	100	100	100	100	100	100	100	100	100	100	100	100	100
M8A1	100	100	100	100	100	0	100	100	100	100	100	100	100	100	100	100	100	100	100	100	100	100	100
M8A4	100	100	100	0	100	0	100	100	100	100	100	100	100	100	100	100	100	100	100	100	100	100	100
ATCC33988	100	100	100	100	100	0	100	0	100	100	100	100	100	100	97	100	100	100	0	100	100	100	100
CHA1	100	100	99,36	0	100	100	100	100	100	100	100	100	100	100	100	100	100	100	100	100	100	100	100
2K-1	100	100	100	0	100	0	100	100	100	100	100	100	100	100	100	100	100	100	100	100	100	100	100
6K-11	100	100	100	0	0	0	100	100	100	100	100	100	100	100	100	100	100	100	100	100	0	100	100
W-101	100	100	100	100	100	0	100	100	100	100	100	100	100	100	100	100	100	100	100	100	100	100	100

Isolate	*motC*	*motD*	*motY*	*mucA*	*mucB*	*mucC*	*mucD*	*mucE*	*mucP*	*pchA*	*pchB*	*pchC*	*pchD*	*pchE*	*pchF*	*pchG*	*pchH*	*pchI*	*pchR*	*pcr1*	*pcr2*	*pcr3*	*pcr4*
PAFIS13_49	100	100	100	99,83	100	100	100	100	100	100	100	100	100	100	100	100	100	100	100	100	100	100	100
IMP66	100	100	100	100	100	100	100	100	100	100	100	100	100	100	100	100	100	100	100	100	100	100	100
ATCC27853	100	100	100	100	100	100	100	100	100	100	100	100	100	100	100	100	100	100	100	100	100	100	100
8D	100	100	100	100	100	100	100	100	100	100	100	100	100	100	100	100	100	100	100	100	100	100	100
2064	100	100	100	100	100	100	100	100	100	100	100	100	100	100	100	100	100	100	100	100	100	100	100
3065	100	100	100	100	100	100	100	100	100	100	100	100	100	100	100	100	100	100	100	100	100	100	100
Pa62222	100	100	100	100	100	100	100	100	100	100	100	100	100	100	100	100	0	100	100	100	100	100	100
L6-1	100	100	100	100	100	100	100	100	100	100	100	100	100	100	100	100	100	100	100	100	100	100	100
DQ8	100	99,89	100	100	100	100	100	100	100	100	100	100	100	100	0	100	100	100	100	100	100	100	100
PA1-Petro	100	100	100	100	100	100	100	100	100	100	100	100	100	100	100	100	100	100	100	100	100	100	100
CMIP8,1	100	100	100	100	100	100	100	100	100	100	100	100	100	100	100	100	100	100	100	100	100	100	100
L100-a	100	100	100	100	100	100	100	100	100	100	100	100	100	100	100	100	100	100	100	100	100	100	100
PAO1	100	100	100	100	100	100	100	100	100	100	100	100	100	100	100	100	100	100	100	100	100	100	100
PSA-2016-06	100	100	100	100	100	100	100	100	100	100	100	100	100	100	82,74	100	100	100	100	100	100	100	100
M8A1	100	100	100	100	100	100	100	100	100	100	100	100	100	100	82,71	100	0	100	100	100	100	100	100
M8A4	100	100	100	100	100	100	100	100	100	100	100	100	100	98,98	99,24	100	100	99,94	100	100	100	100	100
ATCC33988	100	100	100	100	100	100	100	100	100	100	100	100	100	0	0	89,71	0	0	100	100	100	100	100
CHA1	100	100	100	100	100	100	100	100	100	100	100	100	100	100	100	100	100	100	100	100	100	100	100
2K-1	100	100	100	100	100	100	100	100	100	100	100	100	100	100	100	100	100	100	100	100	100	100	100
6K-11	100	100	0	100	100	100	100	100	100	0	0	0	0	0	0	0	0	0	0	100	100	100	100
W-101	100	100	100	100	100	100	100	100	100	100	100	100	100	100	100	100	100	100	100	100	100	100	100

Isolate	*pcrD*	*pcrG*	*pcrH*	*pcrR*	*pcrV*	*phzA1*	*phzB1*	*phzC1*	*phzD1*	*phzE1*	*phzF1*	*phzG1*	*phzH*	*phzM*	*phzS*	*pilA*	*pilB*	*pilC*	*pilE*	*pilF*	*pilG*	*pilH*	*pilI*
PAFIS13_49	100	100	100	100	100	100	100	0	100	0	100	100	100	100	100	97,78	100	100	100	100	100	100	100
IMP66	100	100	100	100	100	100	100	100	100	100	100	100	100	100	100	97,78	100	0	100	100	100	100	100
ATCC27853	100	100	100	100	100	100	100	100	100	100	100	100	100	100	100	0	100	0	100	100	100	100	100
8D	100	100	100	100	100	100	100	100	100	100	100	100	100	100	100	0	100	100	100	100	100	100	100
2064	100	100	100	100	100	100	100	0	0	0	0	100	100	100	100	0	100	100	100	100	100	100	100
3065	100	100	100	100	100	100	100	0	0	0	0	100	100	100	100	0	100	100	100	100	100	100	100
Pa62222	100	100	100	100	100	100	100	100	100	100	100	100	100	100	100	0	100	100	100	100	100	100	100
L6-1	100	100	100	100	100	100	100	100	100	100	100	100	100	100	100	0	100	100	100	100	100	100	100
DQ8	100	100	100	100	100	100	100	100	100	100	100	100	100	100	100	0	100	100	100	100	100	100	100
PA1-Petro	100	100	100	100	100	100	98,36	100	100	100	100	100	100	100	100	0	99,88	0	0	100	100	100	100
CMIP8,1	100	100	100	100	100	100	98,36	0	100	100	100	100	100	100	100	0	99,88	0	0	100	100	100	100
L100-a	100	100	100	100	100	100	100	100	100	100	100	100	100	100	100	0	99,88	0	0	100	100	100	100
PAO1	100	100	100	100	100	100	100	100	100	100	100	100	100	100	100	100	100	100	100	100	100	100	100
PSA-2016-06	100	100	100	100	100	100	100	100	100	100	100	100	100	100	100	97,78	100	100	100	100	100	100	100
M8A1	82,51	100	100	100	100	100	100	100	0	90,23	100	100	100	100	100	97,78	100	100	100	100	100	100	100
M8A4	100	100	100	100	100	90,59	100	100	100	100	100	100	100	100	100	0	100	0	100	100	100	100	100
ATCC33988	100	100	100	100	100	100	100	100	100	100	100	100	100	100	100	0	100	0	100	100	100	100	100
CHA1	100	100	100	100	100	100	100	100	100	100	100	100	100	100	100	0	100	98,67	100	100	100	100	100
2K-1	100	100	100	100	100	100	100	100	100	100	100	100	100	100	100	0	99,88	0	0	100	100	100	100
6K-11	100	100	100	100	100	90,59	88,96	0	0	0	0	0	100	100	0	0	99,88	0	0	100	100	100	100
W-101	100	100	100	100	100	0	0	100	100	88,27	100	0	100	100	100	0	100	0	100	100	100	100	100

Isolate	*pilJ*	*pilK*	*pilM*	*pilN*	*pilO*	*pilP*	*pilQ*	*pilR*	*pilS*	*pilT*	*pilU*	*pilV*	*pilW*	*pilX*	*pilY1*	*pilY2*	*plcH*	*popB*	*popD*	*popN*	*ppkA*	*pppA*	*pscB*	*pscC*	*pscD*	*pscE*
PAFIS13_49	100	100	100	100	100	100	100	100	100	100	100	100	100	100	99,66	100	100	100	100	100	100	100	100	100	100	100
IMP66	100	100	100	100	100	100	100	100	100	100	100	100	100	100	99,66	100	100	100	100	100	100	100	100	100	100	100
ATCC27853	100	100	100	100	100	100	100	100	100	100	100	100	100	100	99,66	100	100	100	100	100	100	100	100	100	100	100
8D	100	100	100	100	100	100	100	100	100	100	100	100	100	100	99,66	100	100	100	100	100	100	100	100	100	100	100
2064	100	100	100	100	100	100	100	100	100	100	100	100	100	100	99,66	100	100	100	100	100	0	100	100	100	100	100
3065	100	100	100	100	100	100	100	100	100	100	100	100	100	100	99,66	100	100	100	100	100	0	100	100	100	100	100
Pa62222	100	100	100	100	100	100	100	100	100	100	100	100	100	100	99,66	100	100	100	100	100	0	100	100	100	100	100
L6-1	100	100	100	100	100	100	100	100	100	100	100	100	100	100	99,66	100	100	99,91	100	100	100	100	100	100	100	100
DQ8	100	100	100	100	100	100	100	100	100	100	100	100	100	100	99,66	100	100	100	100	100	100	100	100	100	100	100
PA1-Petro	100	100	100	100	100	100	99,44	100	100	100	100	0	0	0	0	0	100	100	100	100	100	100	100	100	100	100
CMIP8,1	100	100	100	100	100	100	99,44	100	100	100	100	0	0	0	0	0	100	100	100	100	100	100	100	100	100	100
L100-a	100	100	100	100	100	100	99,44	100	100	100	100	0	0	0	0	0	100	100	100	100	100	100	100	100	100	100
PAO1	100	100	100	100	100	100	100	100	100	100	100	100	100	100	100	100	100	100	100	100	100	100	100	100	100	100
PSA-2016-06	100	100	100	100	100	100	100	100	100	100	100	100	100	100	99,66	100	100	100	100	100	100	100	100	100	100	100
M8A1	100	100	100	100	100	100	100	100	100	100	100	100	100	100	99,66	100	100	100	100	100	0	100	100	100	100	100
M8A4	100	100	100	100	100	100	100	100	100	100	100	100	100	100	99,66	100	100	100	100	100	0	100	100	100	100	100
ATCC33988	100	100	100	100	100	100	100	100	100	100	100	100	100	100	99,66	100	100	100	100	100	0	100	100	100	100	100
CHA1	100	100	100	100	100	100	100	100	100	100	100	100	100	100	99,66	100	100	100	100	100	100	100	100	100	100	100
2K-1	100	100	100	100	100	100	99,44	100	100	100	100	0	0	0	0	0	100	100	100	100	100	100	100	100	100	100
6K-11	100	100	100	100	100	100	99,44	100	100	100	100	0	0	0	0	0	100	100	100	100	100	100	100	100	100	100
W-101	100	100	100	100	100	100	100	100	100	100	100	100	100	100	99,43	100	100	100	100	100	100	100	100	100	100	100

Isolate	*pscF*	*pscG*	*pscH*	*pscI*	*pscJ*	*pscK*	*pscL*	*pscN*	*pscO*	*pscP*	*pscQ*	*pscR*	*pscS*	*pscT*	*pscU*	*ptxR*	*pvcA*	*pvcB*	*pvcC*	*pvcD*	*pvdA*	*pvdD*	*pvdE*	*pvdF*
PAFIS13_49	100	100	100	100	100	99,40	100	100	100	100	100	100	100	100	100	100	100	99,89	100	100	100	0	0	99,15
IMP66	100	100	100	100	100	99,40	100	100	100	96,22	100	100	100	100	100	100	100	100	100	100	100	0	0	99,15
ATCC27853	100	100	100	100	100	99,40	100	100	100	97,3	100	100	100	100	100	100	100	100	100	100	100	0	0	100
8D	100	100	100	100	100	99,40	100	100	100	98,38	100	100	100	100	100	100	100	100	100	100	100	100	100	100
2064	100	100	100	100	100	99,40	100	0	100	0	100	100	100	100	100	100	100	100	100	100	100	0	100	100
3065	100	100	100	100	100	99,40	100	0	100	0	100	100	100	100	100	100	100	100	100	100	100	0	100	100
Pa62222	100	100	100	100	100	99,40	100	100	100	0	100	100	100	100	100	100	100	89,84	100	100	100	0	100	100
L6-1	100	100	100	100	100	99,40	100	100	100	97,3	100	100	100	100	100	100	100	100	99,93	100	100	100	100	100
DQ8	100	100	100	100	100	99,40	100	100	100	97,3	100	100	100	100	100	100	100	100	100	100	98,35	0	100	100
PA1-Petro	100	100	100	100	100	99,40	100	100	100	98,38	100	100	100	100	100	100	100	100	100	99,85	100	0	0	100
CMIP8,1	100	100	100	100	100	99,40	100	100	100	98,38	100	100	100	100	100	100	100	100	100	99,85	100	0	0	100
L100-a	100	100	100	100	100	99,40	100	100	100	97,84	100	100	100	100	100	100	100	100	100	99,85	100	0	0	100
PAO1	100	100	100	100	100	100,00	100	100	100	100	100	100	100	100	100	100	100	100	100	100	100	100	100	100
PSA-2016-06	100	100	100	100	100	99,40	100	100	100	100	100	100	100	100	100	100	100	100	100	100	100	0	0	99,15
M8A1	100	100	100	100	100	99,40	100	100	100	97,84	100	100	100	100	100	100	100	91,78	100	100	100	0	0	99,15
M8A4	100	100	100	100	100	99,40	100	100	100	0	100	100	100	100	100	100	100	100	100	100	100	0	0	99,15
ATCC33988	100	100	100	100	100	99,40	100	100	100	98,92	100	100	100	100	100	100	100	100	100	100	100	0	0	99,15
CHA1	100	100	100	100	100	99,40	100	100	100	100	100	100	100	100	100	100	100	100	100	99,85	100	100	100	100
2K-1	100	100	100	100	100	99,40	100	100	100	98,38	100	100	100	100	100	100	100	100	100	100	100	0	0	100
6K-11	100	100	100	100	100	99,40	100	100	100	100	100	100	100	100	100	0	0	0	0	0	100	0	0	100
W-101	100	100	100	100	100	99,40	100	100	100	97,84	100	100	100	100	100	100	100	100	100	100	100	0	0	100

Isolate	*pvdG*	*pvdH*	*pvdI*	*pvdJ*	*pvdL*	*pvdM*	*pvdN*	*pvdO*	*pvdP*	*pvdQ*	*pvdS*	*rhlA*	*rhlB*	*rhlC*	*rhlI*	*tagF/ pppB*	*tagQ*	*tagR*	*tagS*	*tagT*	*toxA*	*tse1*	*tse2*	*tse3*
PAFIS13_49	100	100	0	0	100	99,7	99,45	100	100	100	100	100	100	100	100	100	100	100	100	100	100	100	100	100
IMP66	100	100	0	0	100	99,7	99,45	100	100	100	100	100	100	100	100	100	100	100	100	100	100	100	100	100
ATCC27853	100	100	0	0	100	99,78	100	100	99,76	100	100	100	100	100	100	100	100	100	100	100	100	100	100	100
8D	100	100	99,98	100	100	100	100	100	100	100	100	100	100	100	100	100	100	100	100	100	100	100	100	100
2064	100	100	0	0	100	100	100	100	100	100	100	100	100	100	100	100	100	100	100	100	100	98,71	100	100
3065	100	100	0	0	100	100	100	100	100	100	100	100	100	100	100	100	100	100	100	100	100	98,71	100	100
Pa62222	100	100	89,12	0	100	100	100	100	100	100	100	100	100	100	100	100	100	100	100	100	100	100	100	100
L6-1	100	100	100	100	100	100	100	100	100	100	100	100	100	100	100	100	100	100	100	100	100	100	100	100
DQ8	100	100	0	0	100	100	100	100	100	100	100	100	100	100	100	100	100	100	100	100	100	100	100	100
PA1-Petro	100	100	0	0	100	100	100	100	100	100	100	100	100	100	100	100	100	100	100	100	100	100	100	100
CMIP8,1	100	100	0	0	100	100	100	100	100	100	100	100	100	100	100	100	100	100	100	100	100	100	100	100
L100-a	100	100	0	0	100	100	100	100	100	100	100	100	100	100	100	100	100	100	100	100	100	100	100	100
PAO1	100	100	100	100	100	100	100	100	100	100	100	100	100	100	100	100	100	100	100	100	100	100	100	100
PSA-2016-06	100	100	0	0	100	99,7	99,45	100	100	100	100	100	100	100	100	100	100	100	100	100	100	100	100	100
M8A1	100	100	0	0	100	99,7	99,45	100	100	100	100	100	100	100	100	100	100	100	100	100	100	100	100	100
M8A4	100	100	0	0	100	99,7	99,45	100	100	100	100	100	100	100	100	100	100	100	100	100	99,53	100	100	100
ATCC33988	100	100	0	0	100	99,7	99,45	100	100	100	100	100	100	100	100	100	100	100	100	100	100	100	100	100
CHA1	100	100	99,98	100	100	100	100	100	100	100	100	100	100	100	100	100	100	100	100	100	100	100	100	100
2K-1	100	100	0	0	100	100	100	100	100	100	100	100	100	100	100	100	100	100	100	100	100	100	100	100
6K-11	0	0	0	0	0	100	100	100	100	100	0	0	0	100	0	100	100	100	100	100	100	100	100	0
W-101	100	100	0	0	100	99,78	100	100	99,76	100	100	100	100	100	100	100	100	100	100	100	100	100	100	100

Isolate	*vgrG1a*	*vgrG1b*	*waaA*	*waaC*	*waaF*	*waaG*	*waaP*	*wzy*	*wzz*	*xcpA/ pilD*	*xcpP*	*xcpQ*	*xcpR*	*xcpS*	*xcpT*	*xcpU*	*xcpV*	*xcpW*	*xcpX*	*xcpY*	*xcpZ*
PAFIS13_49	100	100	100	100	100	100	100	0	0	100	100	100	100	100	100	100	100	100	100	100	100
IMP66	100	100	100	100	100	100	100	0	0	100	100	100	100	100	100	100	100	100	100	100	100
ATCC27853	100	100	100	100	100	100	100	0	0	100	100	100	100	100	100	100	100	100	100	100	100
8D	100	100	100	100	100	100	100	100	100	100	100	100	100	100	100	100	100	98,74	100	100	100
2064	100	0	100	100	100	100	100	100	100	100	100	100	100	100	100	100	100	98,74	100	100	100
3065	100	0	100	100	100	100	100	100	100	100	100	100	100	100	100	100	100	98,74	100	100	100
Pa62222	100	85,18	100	100	100	100	100	100	100	100	100	100	100	100	100	100	100	98,74	100	100	100
L6-1	100	100	100	100	100	100	100	100	100	100	100	100	100	100	100	100	100	98,74	100	100	100
DQ8	100	0	99,92	100	100	100	100	100	100	100	100	100	100	100	100	100	100	98,74	99,8	100	100
PA1-Petro	100	100	100	100	100	100	100	0	0	99,31	100	99,85	100	100	100	100	100	100	100	100	100
CMIP8,1	100	100	100	100	100	100	100	0	0	99,31	100	99,85	100	100	100	100	100	100	100	100	100
L100-a	100	100	100	100	100	100	100	0	0	99,31	100	99,85	100	100	100	100	100	100	100	100	100
PAO1	100	100	100	100	100	100	100	100	100	100	100	100	100	100	100	100	100	100	100	100	100
PSA-2016-06	100	100	100	100	100	100	100	0	0	100	100	100	100	100	100	100	100	100	100	100	100
M8A1	100	100	100	100	100	100	100	0	0	100	100	100	100	100	100	100	100	100	100	100	100
M8A4	100	100	100	100	100	100	100	0	0	100	100	99,85	100	100	100	100	100	100	100	100	100
ATCC33988	100	100	100	100	100	100	100	0	0	100	100	100	100	100	100	100	100	100	100	100	100
CHA1	100	0	100	100	100	100	100	0	0	100	100	99,85	100	100	100	100	100	100	100	100	100
2K-1	100	0	100	100	100	100	100	0	0	98,63	100	100	100	100	100	100	100	98,74	100	100	100
6K-11	100	100	100	100	100	100	100	0	0	98,63	100	100	100	100	100	100	100	98,74	100	100	100
W-101	100	100	100	100	100	100	100	0	0	100	100	99,85	100	100	100	100	100	100	100	100	100

Supplementary Table S3. ARGs detected by ResFinder in *P. aeruginosa* strains

**Strain**	**MLST Sequence Type**	**Group**	***aph(3')-IIb***	***aph(3')-Ia***	***bla* $_{OXA-50}$ variant**	***bla* $_{PDC}$ variant**	***catB7***	***crpP***	***fosA***
PAFIS13_49	ST132	clinical	100	-	100	100	100	100	100
IMP66	ST132	environmental	100	-	100	100	100	100	100
ATCC27853	ST155	clinical	100	-	100	100	100	100	100
8D	ST244	environmental	100	-	100	100	100	-	100
2064	ST244	clinical	100	-	100	100	100	100	100
3065	ST244	clinical	100	-	100	100	100	100	100
Pa62222	ST244	clinical	100	-	100	100	100	100	100
L6-1	ST267	environmental	100	-	100	100	100	100	100
DQ8	ST267	environmental	100	100	100	100	100	100	100
PA1-Petro	ST532	environmental	100	-	100	100	100	100	100
CMIP8.1	ST532	environmental	100	-	100	100	100	100	100
L00-a	ST532	clinical	100	-	100	100	100	100	100
PAO1	ST549	clinical	100	-	100	100	100	-	100
PSA-2016-06	ST918	clinical	100	-	100	100	100	100	100
M8A1	ST918	environmental	100	-	100	100	100	-	100
M8A4	ST1054	environmental	100	-	100	100	100	-	100
ATCC33988	ST1232	environmental	100	-	100	100	97,18	-	100
CHA1	ST1503	environmental	100	-	100	100	100	100	100
2K-1	ST4371	environmental	100	-	100	100	100	100	100
6K-11	ST4371	environmental	100	-	100	100	100	100	100
W-101	ST4655	environmental	100	-	100	100	100	-	100

Supplementary Figure 1. showing alignment of the protein sequences of AlkB1 **(A)** and AlkB2 **(B)** enzymes of *P. aeruginosa* strains CHA1 and PAO1, respectively.

Supplementary Figure 1A.

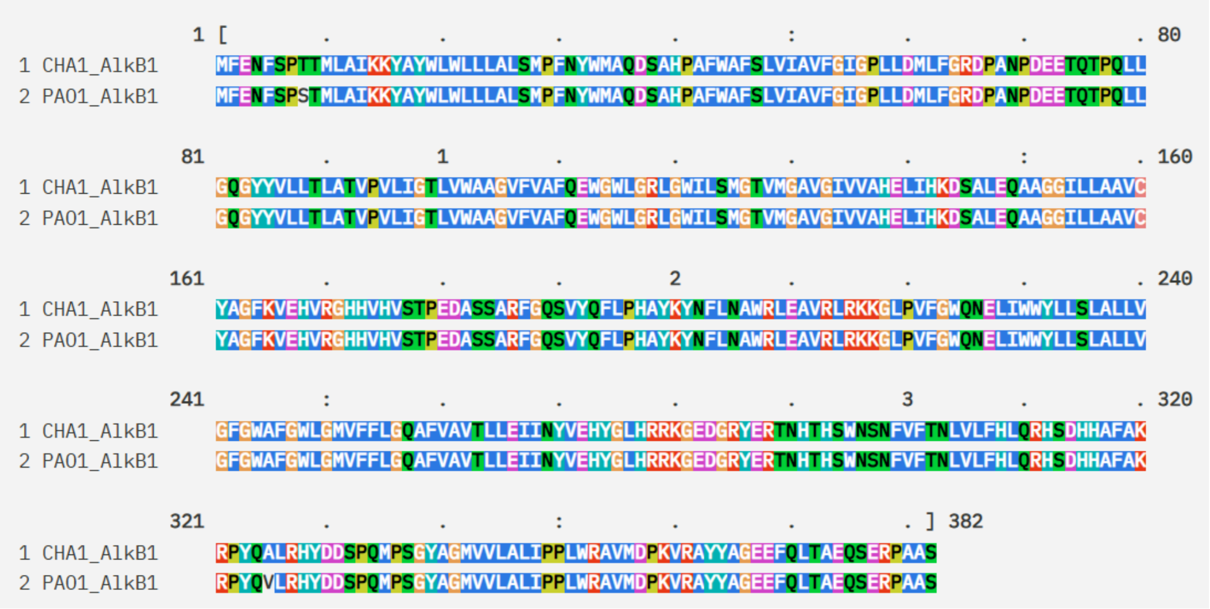

Supplementary Figure 1B.

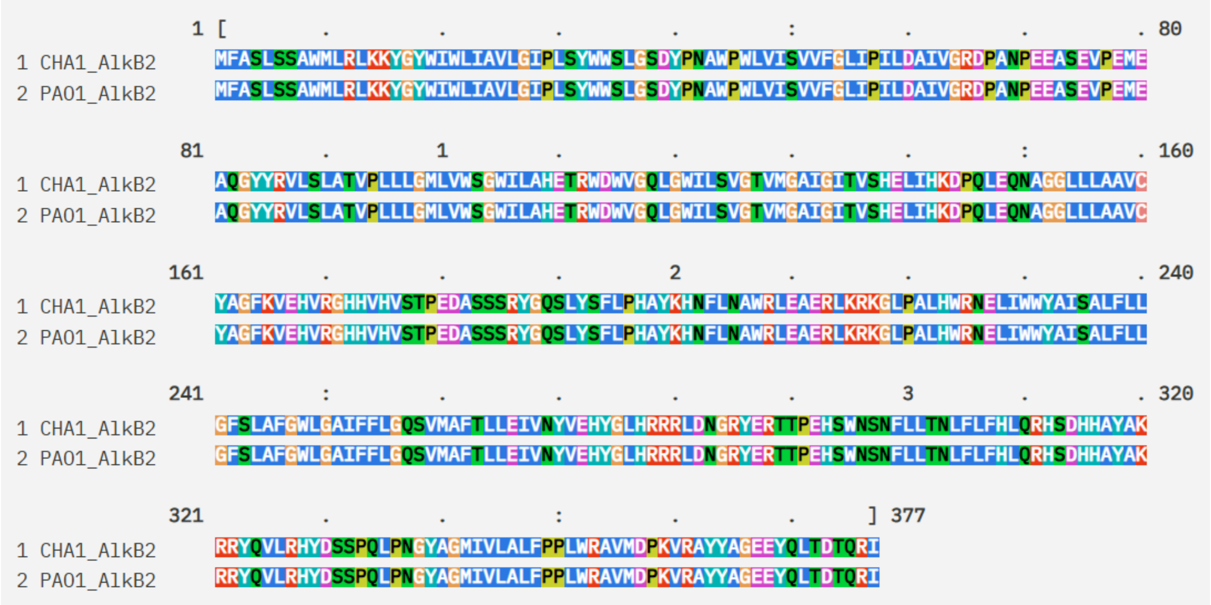

The CLUSTAL colour scheme was used for generating the alignment.

SUPPLEMENTARY FIGURE 2.

Supplementary Figure 2A. Multiple alignment of the protein sequences encoded by the *mexR* genes of *P. aeruginosa* isolates. V126E amino acid substitutions compared to MexR of the PAO1 strain are indicated by a black arrow.

```
mexR_W-101        MNYPVNPDLMPALMAVFQHVRTRIQSELDCQRLDLTPPDVHVLKLIDEQRGLNLQDLGRQ   60
mexR_M8A4         MNYPVNPDLMPALMAVFQHVRTRIQSELDCQRLDLTPPDVHVLKLIDEQRGLNLQDLGRQ   60
mexR_M8A1         MNYPVNPDLMPALMAVFQHVRTRIQSELDCQRLDLTPPDVHVLKLIDEQRGLNLQDLGRQ   60
mexR_L6-1         MNYPVNPDLMPALMAVFQHVRTRIQSELDCQRLDLTPPDVHVLKLIDEQRGLNLQDLGRQ   60
mexR_IMP66        MNYPVNPDLMPALMAVFQHVRTRIQSELDCQRLDLTPPDVHVLKLIDEQRGLNLQDLGRQ   60
mexR_DQ8          MNYPVNPDLMPALMAVFQHVRTRIQSELDCQRLDLTPPDVHVLKLIDEQRGLNLQDLGRQ   60
mexR_ATCC33988    MNYPVNPDLMPALMAVFQHVRTRIQSELDCQRLDLTPPDVHVLKLIDEQRGLNLQDLGRQ   60
mexR_PAO1         MNYPVNPDLMPALMAVFQHVRTRIQSELDCQRLDLTPPDVHVLKLIDEQRGLNLQDLGRQ   60
mexR_ATCC27853    MNYPVNPDLMPALMAVFQHVRTRIQSELDCQRLDLTPPDVHVLKLIDEQRGLNLQDLGRQ   60
mexR_6K-11        MNYPVNPDLMPALMAVFQHVRTRIQSELDCQRLDLTPPDVHVLKLIDEQRGLNLQDLGRQ   60
mexR_2K-1         MNYPVNPDLMPALMAVFQHVRTRIQSELDCQRLDLTPPDVHVLKLIDEQRGLNLQDLGRQ   60
mexR_PA1-Petro    MNYPVNPDLMPALMAVFQHVRTRIQSELDCQRLDLTPPDVHVLKLIDEQRGLNLQDLGRQ   60
mexR_CMIP8.1      MNYPVNPDLMPALMAVFQHVRTRIQSELDCQRLDLTPPDVHVLKLIDEQRGLNLQDLGRQ   60
mexR_CHA1         MNYPVNPDLMPALMAVFQHVRTRIQSELDCQRLDLTPPDVHVLKLIDEQRGLNLQDLGRQ   60
                  ************************************************************

mexR_W-101        MCRDKALITRKIRELEGRNLVRRERNPSDQRSFQLFLTDEGLAIHQHAEAIMSRVHDELF   120
mexR_M8A4         MCRDKALITRKIRELEGRNLVRRERNPSDQRSFQLFLTDEGLAIHQHAEAIMSRVHDELF   120
mexR_M8A1         MCRDKALITRKIRELEGRNLVRRERNPSDQRSFQLFLTDEGLAIHQHAEAIMSRVHDELF   120
mexR_L6-1         MCRDKALITRKIRELEGRNLVRRERNPSDQRSFQLFLTDEGLAIHQHAEAIMSRVHDELF   120
mexR_IMP66        MCRDKALITRKIRELEGRNLVRRERNPSDQRSFQLFLTDEGLAIHQHAEAIMSRVHDELF   120
mexR_DQ8          MCRDKALITRKIRELEGRNLVRRERNPSDQRSFQLFLTDEGLAIHQHAEAIMSRVHDELF   120
mexR_ATCC33988    MCRDKALITRKIRELEGRNLVRRERNPSDQRSFQLFLTDEGLAIHQHAEAIMSRVHDELF   120
mexR_PAO1         MCRDKALITRKIRELEGRNLVRRERNPSDQRSFQLFLTDEGLAIHQHAEAIMSRVHDELF   120
mexR_ATCC27853    MCRDKALITRKIRELEGRNLVRRERNPSDQRSFQLFLTDEGLAIHQHAEAIMSRVHDELF   120
mexR_6K-11        MCRDKALITRKIRELEGRNLVRRERNPSDQRSFQLFLTDEGLAIHQHAEAIMSRVHDELF   120
mexR_2K-1         MCRDKALITRKIRELEGRNLVRRERNPSDQRSFQLFLTDEGLAIHQHAEAIMSRVHDELF   120
mexR_PA1-Petro    MCRDKALITRKIRELEGRNLVRRERNPSDQRSFQLFLTDEGLAIHQHAEAIMSRVHDELF   120
mexR_CMIP8.1      MCRDKALITRKIRELEGRNLVRRERNPSDQRSFQLFLTDEGLAIHQHAEAIMSRVHDELF   120
mexR_CHA1         MCRDKALITRKIRELEGRNLVRRERNPSDQRSFQLFLTDEGLAIHQHAEAIMSRVHDELF   120
                  ************************************************************

mexR_W-101        APLTPVEQATLVHLLDQCLAAQPLEDI 147
mexR_M8A4         APLTPVEQATLVHLLDQCLAAQPLEDI 147
mexR_M8A1         APLTPVEQATLVHLLDQCLAAQPLEDI 147
mexR_L6-1         APLTPVEQATLVHLLDQCLAAQPLEDI 147
mexR_IMP66        APLTPVEQATLVHLLDQCLAAQPLEDI 147
mexR_DQ8          APLTPVEQATLVHLLDQCLAAQPLEDI 147
mexR_ATCC33988    APLTPVEQATLVHLLDQCLAAQPLEDI 147
mexR_PAO1         APLTPVEQATLVHLLDQCLAAQPLEDI 147
mexR_ATCC27853    APLTPVEQATLVHLLDQCLAAQPLEDI 147
mexR_6K-11        APLTPVEQATLVHLLDQCLAAQPLEDI 147
mexR_2K-1         APLTPVEQATLVHLLDQCLAAQPLEDI 147
mexR_PA1-Petro    APLTPEEQATLVHLLDQCLAAQPLEDI 147
mexR_CMIP8.1      APLTPEEQATLVHLLDQCLAAQPLEDI 147
mexR_CHA1         APLTPEEQATLVHLLDQCLAAQPLEDI 147
                  ***** *********************
```

V126E

Supplementary Figure 2B. Multiple alignment of the protein sequences encoded by the *nalC* genes of *P. aeruginosa* isolates. G71E and S209R amino acid substitutions compared to NalC of the PAO1 strain are indicated by black arrows.

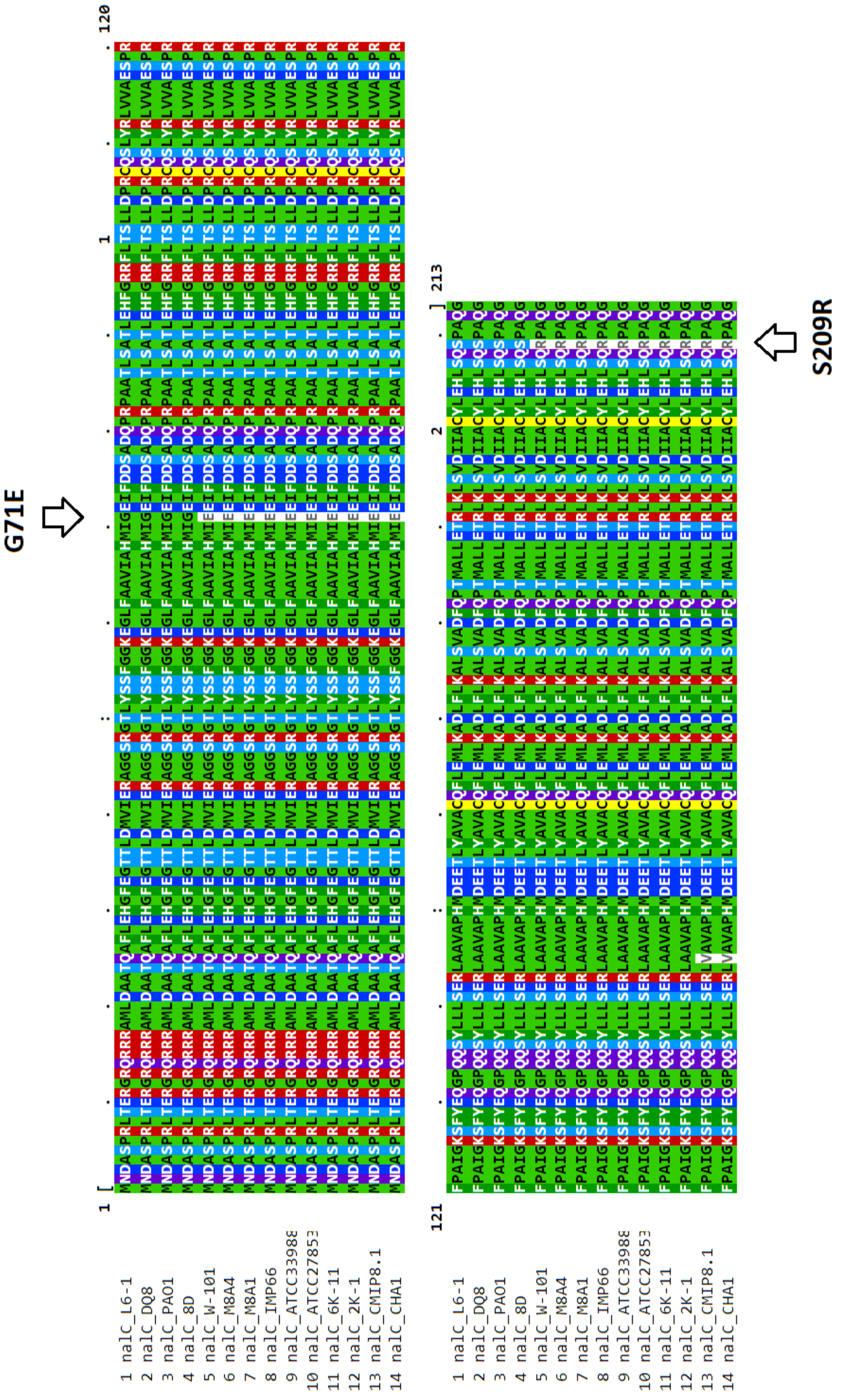

Supplementary Figure S3. Clustering of *P. aeruginosa* isolates based on virulence factors detected in WGS data

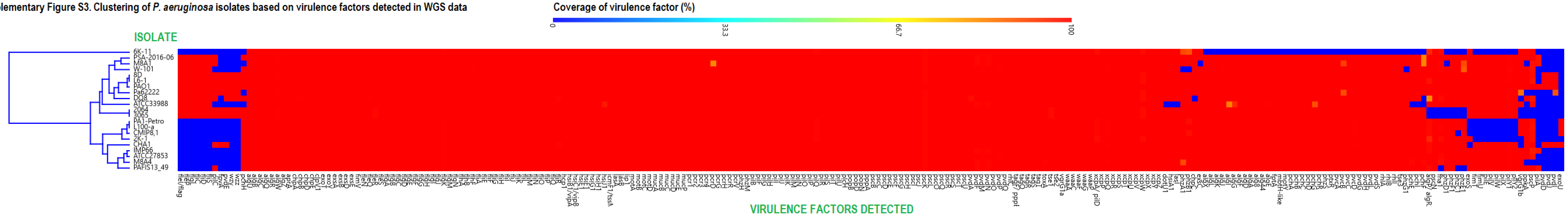

Supplement: Supplementary file 1 [file microorganisms-13-00688-s001.zip › microorganisms-3504633-supplementary.pdf]
